# Approximate Bayesian computation for inferring Waddington landscapes from single-cell data

**DOI:** 10.1098/rsos.231697

**Published:** 2024-07-10

**Authors:** Yujing Liu, Stephen Y. Zhang, Istvan T. Kleijn, Michael P. H. Stumpf

**Affiliations:** ^1^ School of Mathematics and Statistics, University of Melbourne, Melbourne, Australia; ^2^ School of BioScience, University of Melbourne, Melbourne, Australia; ^3^ Institute of Cancer Research, London, UK

**Keywords:** epigenetic landscape, likelihood free inference, quasi-potential

## Abstract

Single-cell technologies allow us to gain insights into cellular processes at unprecedented resolution. In stem cell and developmental biology snapshot data allow us to characterize how the transcriptional states of cells change between successive cell types. Here, we show how approximate Bayesian computation (ABC) can be employed to calibrate mathematical models against single-cell data. In our simulation study, we demonstrate the pivotal role of the adequate choice of distance measures appropriate for single-cell data. We show that for good distance measures, notably optimal transport with the Sinkhorn divergence, we can infer parameters for mathematical models from simulated single-cell data. We show that the ABC posteriors can be used (i) to characterize parameter sensitivity and identify dependencies between different parameters and (ii) to construct representations of the Waddington or epigenetic landscape, which forms a popular and interpretable representation of the developmental dynamics. In summary, these results pave the way for fitting mechanistic models of stem cell differentiation to single-cell data.

## 1. Introduction

While developmental biology has largely progressed through observational studies, from the beginning of the twentieth century this seemingly intricate, bewilderingly complex, yet robust, process has also fascinated mathematicians.

Of particular importance has been C. H. Waddington’s epigenetic landscape [1]. Originally intended as a metaphor for developmental processes, the idea of a landscape has continued to guide thinking among biologists and mathematicians; the Fields medalist René Thom, for example, was interested in developmental biology as a potential manifestation of catastrophe theory.

Over the past decade, there has been a resurgence in interest from mathematicians, developmental biologists and bioengineers in the epigenetic or Waddington landscape. We can use it as a mathematical and computational tool to reason about and even predict cell fates [2]. Recent studies [3–6] have given us qualitative insights into the fundamental dynamics underlying differentiation at the cellular level. In a deterministic framework, it was shown that even fundamental developmental dynamics can be understood in terms of elegant mathematical models of small gene regulation networks [5]. But to account for the (experimentally quantifiable) randomness prevailing among sub-cellular molecular processes, we must extend such analyses to incorporate stochastic processes. This has become especially important because stochastic effects can change the dynamics not just quantitatively but qualitatively, profoundly reshaping the manifold on which dynamics occur. Specifically, multiplicative noise can destroy or created cell states that are defined in terms of the attracting states of the determinisic system [6,7].

While the mathematical analysis of dynamical systems has achieved maturity, our ability to challenge mathematical models of developmental systems with data is lagging behind both the mathematical theory and our capability to probe developmental systems in experiment. Technological advances in single-cell biology provide us with exquisitely detailed snapshots of the transcriptomic states of single cells [8]; and before long we will also be able to collect single-cell protein data of the required quality and quantity [9,10]. And while descriptive and statistical analyses of single-cell data have progressed in lock-step with experimental technologies and new data, mechanistic modelling in light of data has progressed more slowly.

Landscapes for dynamical system models of developmental systems are typically formulated through thermodynamic approaches with detailed chemical assumptions about kinetics of gene transcription factors [11]. But the inference of parameters underpinning the landscapes remains an open problem. It is well known that variation of the parameters of a dynamical system [12] can lead to changes in the cellular state or even the structure of the landscapes: for example, at some critical value of parameters, creation or destruction of attracting states may occur which leads to different landscape structures. Such changes in dynamical systems due to the variation of parameters are known as bifurcations [3,13] and are a central phenomenon of study in the dynamical systems literature. Therefore, to reconstruct the landscapes that agree with experimental observations, it is necessary to have parameters adequately inferred.

Coupling single-cell data to modelling has been challenging as it has been difficult to, for example, estimate reaction rate parameters for dynamical systems models. Here, we explore the use of approximate Bayesian computation (ABC) [14] as a tool to estimate parameters for dynamical systems describing stem cell differentiation. In cases where conventional likelihood-based approaches fail or are difficult to apply because the likelihood is intractable, ABC methods can provide (approximate) answers.

Most current single-cell data provide snapshots of the expression state of a system. Temporal information can typically only be gleaned indirectly, and a host of statistical approaches have been used to remedy this situation [15]. Each of these methods has, however, associated degrees of uncertainty. Here, we take a complementary approach where data are assumed to have been generated by a stochastic dynamical system (at different times). We show below that this approach can be used to infer model parameters and determine parameter sensitivities for models of stem cell differentiation. We show that the manner in which we summarize the data—or calculate distances between observed and simulated data—affects the efficiency and reliability of parameter inference profoundly.

## 2. Methods

### 2.1. Waddington landscapes and quasi-potentials for developmental processes

Mathematically, we can characterize the evolution of cell states, *X*_*t*_, over time *t* by a stochastic differential equation (SDE),

2.1 $$dX_{t}=\mu (X_{t};\theta )\;dt+\sigma (X_{t};\theta )\;dW_{t},$$

where *μ*(*X*_*t*_; *θ*)d*t* captures the deterministic dynamics of our system parametrized by *θ* and *σ*(*X*_*t*_; *θ*)d*W*_*t*_ depicts the random variations or stochastic properties of this system parametrized by *θ*.

In many cases, the focus has been on gradient systems, where we have a potential function, *U*(*X*), such that

2.2 $$\frac{dX_{t}}{dt}=\mu +\sigma \cdot \xi (t)=-\nabla U(X_{t})+\sigma \cdot \xi (t),$$

where *ξ*(*t*) is the white noise with *E*(*ξ*(*t*)) = 0, *E*(*ξ*(*t*)*ξ*(*s*)) = *δ*(*t* − *s*). The potential function *U*(*X*_*t*_) describes the deterministic dynamics of cell states at each time point and determines the attracting states. The choice of the stochastic dynamics can distort this potential profoundly.

While gradient dynamical systems are flexible and popular—they have also been argued to capture hall-marks of more general developmental systems inspired by developmental biology—they cannot describe all aspects, especially oscillatory and clock-like processes, including the cell cycle [16,17]. We nevertheless seek to capture many of the dynamics in terms of landscape descriptions, but we make the approximate nature explicit by referring to the mathematical description as the quasi-potential, $\tilde{U}(X)$.

We generally cannot determine *U*(*X*) or $\tilde{U}(t)$ analytically and instead rely on simulations. We can determine the approximate quasi-potential via the probability density function *p*(*X*_*t*_), which contains the information about how probable state *X*_*t*_ is. The quasi-potential is then given as [17]

2.3 $$\tilde{U}(X)=-\log p(X).$$

The quasi-potential is thus the logarithm of the stationary probability distribution that a cell is in state *X* [18]. The agreement between quasi-potential and true or deterministic potential depends heavily on the coefficient of noise term. When coupled with additive noise (*σ*(*X*_*t*_; *θ*) = *c*), the true potential can always be recovered by the quasi-potential. However, when dealing with multiplicative noise (i.e. *σ*(*X*_*t*_; *θ*) depends on the state *X*_*t*_), there would be large deviation of quasi-potential and true potential [6].

### 2.2. ABC and ABC–SMC

In a Bayesian framework, we estimate the posterior probability of a model’s parameters via

P(θ|D)∝f (D|θ)P(θ),

where *f*(*D*|*θ*) is the likelihood of *θ* given the data *D* and where *P*(*θ*) is the prior for the model parameters, *θ*. In ABC, the calculation of the likelihood is replaced by a comparison between the observed data and simulated data. ABC methods have the following generic form [19]:

**Algorithm 1.** ABC rejection algorithm.

**Require:** ϵ>0, N>0
i←0
**while** i<N **do**
$\theta^{*}∼P(\theta )$. ⊳ Sample a candidate parameter $\theta^{*}$ from proposal distribution P(θ)
$X∼f\;(D|\theta^{*})$ ⊳ Simulate a dataset X from the model with parameter $\theta^{*}$
**if** d(D, X)≤ϵ **then**
$\theta_{i}\leftarrow \theta^{*}$ ⊳ accept $\theta^{*}$
i←i+1
**end if**
**end while**

where *d* is a distance function and the tolerance ϵ>0 defines the intended degree of alignment between *D* and *X*.

The result of any ABC inference is a set of parameter samples from the approximate posterior distribution P(θ|d(D,X)≤ϵ). When ϵ is sufficiently small then the distribution P(θ|d(D,X)≤ϵ) can be regarded as a reliable approximation for the ‘actual’ posterior distribution *P*(*θ*|*D*) [19].

The basic algorithm outlined above, known as ABC rejection, is typically too slow for any problem of interest with more than a few parameters. A range of improvements have been developed in the literature, including Markov chain Monte Carlo, and sequential Monte Carlo (SMC) approaches. Here, we use the SMC approach of Toni *et al*. [14] adapted to the case of single-cell data obtained from simulations of dynamical systems as the data collected from true developmental systems. In this case, a key insight is to find a good distance appropriate for single-cell data. We will discuss this in the next section.

### 2.3. Developmental model and simulation procedure

**Figure 1.**
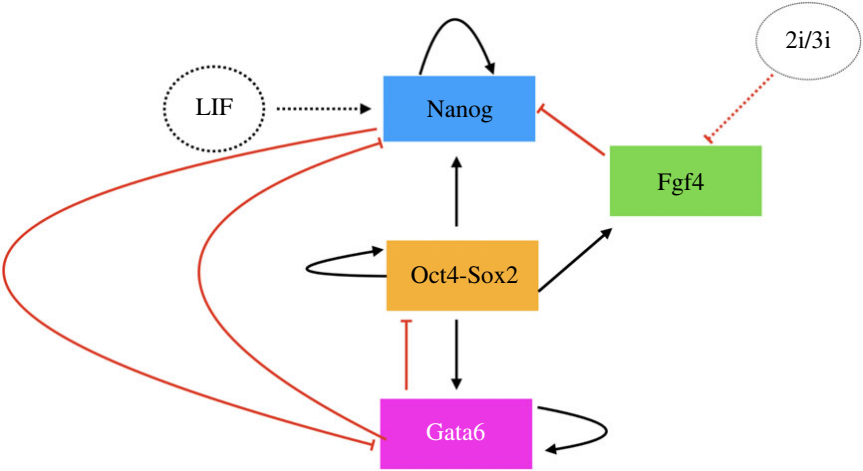
The transcription factor interaction relationship circuit with external factors influences where red line stands for inhibition and black line stands for promotion.

Our analysis will focus on a model that has been used to model embryonic stem cell differentiation processes [20], see figure 1, where each line represents nonlinear inhibition or promotion among these factors. In specific, this model is characterized by the temporal dynamics of four transcription factors, Nanog (N), Oct4-Sox2 (O), Fgf4 (F) and Gata6 (G), where Nanog, Oct4 and Sox2 are complex for maintaining pluripotency and Gata6 is a standard bio-marker for cell differentiation. The detailed representation of their relationship is shown in figure 1 where each line represents nonlinear inhibition or promotion among these factors.

We will model the stochastic feature of this process by implementing a Wiener process on each factor [21].

**Figure 2.**
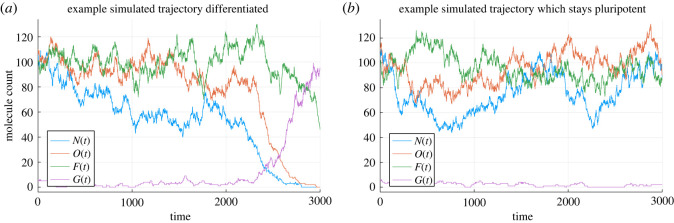
The simulated sample trajectories of the four key transcription factors over time. The left one is Nanog stays pluripotent and the right one is Nanog differentiated into Gata6.

This system gives rise to two distinct cell states: (i) the pluripotent state is characterized by high values of Nanog and low levels of Gata6; (ii) the differentiated state is characterized by the opposite behaviours for both genes (figure 2). The deterministic part of the system is given in [16,20,21]:

2.4 $$\begin{array}{rl} & \frac{d[N]}{dt}=\frac{k_{0}[O](c_{0}+c_{1}{\left[N\right]}^{2}+k_{0}[O]+c_{2}LIF)}{1+(k_{0}[O](c_{1}{\left[N\right]}^{2}+k_{0}[O]+c_{2}LIF+c_{3}{\left[F\right]}^{2}))+c_{4}[O]{\left[G\right]}^{2}}-\gamma [N],\\ & \frac{d[O]}{dt}=\alpha +\frac{e_{0}+e_{1}[O]}{1+e_{1}[O]+e_{2}{\left[G\right]}^{2}}-\gamma [O],\\ & \frac{d[F]}{dt}=\frac{a_{0}+a_{1}[O]}{1+a_{1}[O]}-\gamma [F]\\ \mathrm{and}\;\; & \frac{d[G]}{dt}=\frac{b_{0}+b_{1}{\left[G\right]}^{2}+b_{3}[O]}{1+b_{1}{\left[G\right]}^{2}+b_{2}{\left[N\right]}^{2}+b_{3}[O]}-\gamma [G], \end{array}\}$$

where the values of parameters we will use in this model, according to Chickarmane [20], are

2.5 $$\begin{array}{cc} & a_{0}=0.01,\;a_{1}=1,\\ & b_{0}=0.005,\;b_{1}=0.005,\;b_{2}=1,\;b_{3}=1,\\ & c_{0}=0.01,\;c_{1}=0.4,\;c_{2}=1,\;c_{3}=0.1,\;c_{4}=0.00135,\\ & e_{0}=0.01,\;e_{1}=1,\;e_{2}=1\\ \mathrm{and}\;\; & k_{0}=0.005,\;\gamma =0.01. \end{array}\}$$

**Figure 3.**
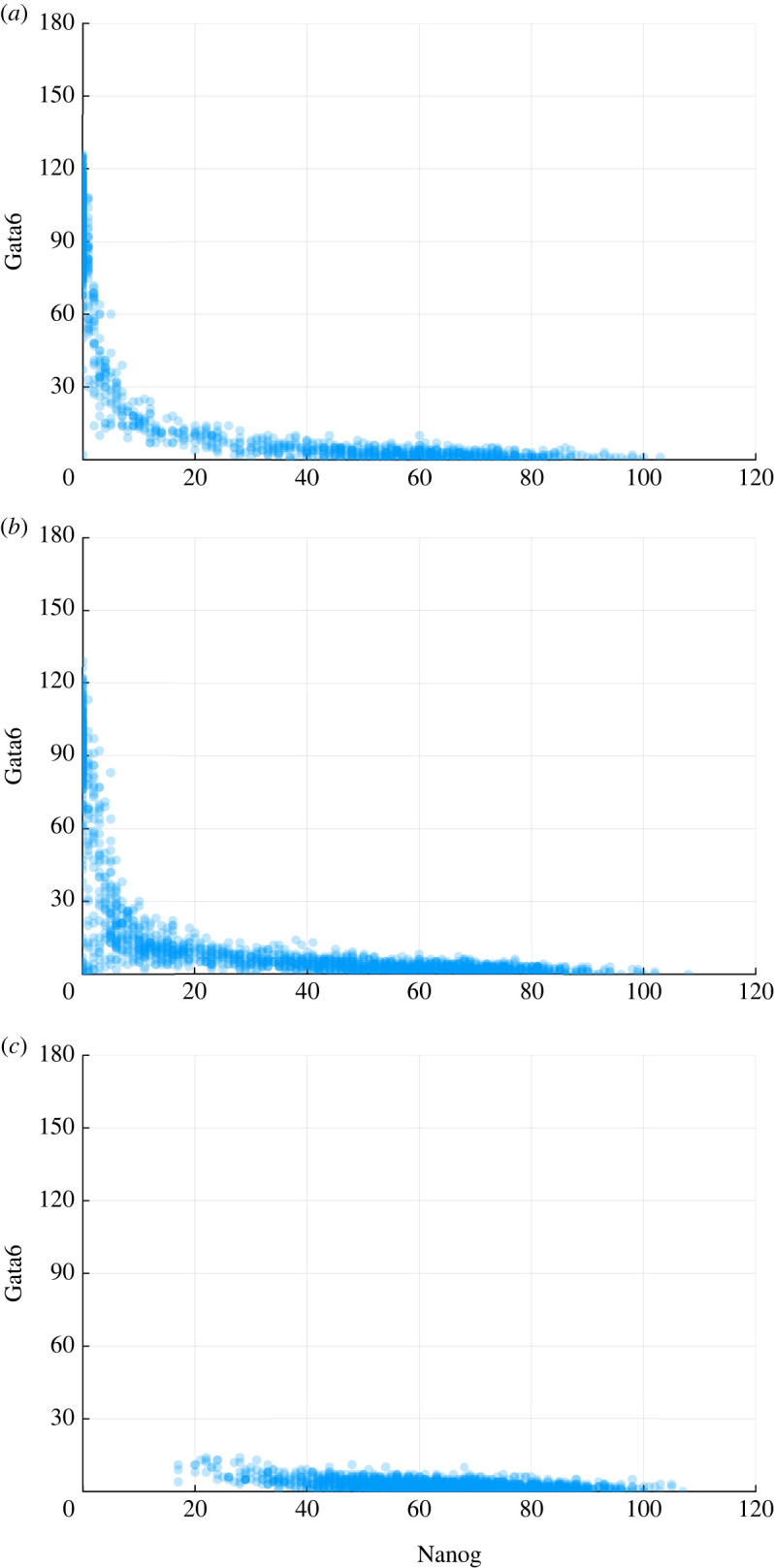
The scatter plots of the relationships between Nanog and Gata6 at the end time for different values of LIF. (*a*) *L* = 5, (*b*) *L* = 50 and (*c*) *L* = 200.

Other than the four transcription factors, there are also some external signals known to influence this system and the propensity functions given above. Leukaemia inhibiting factor (LIF) is a cytokine known to inhibit the cell differentiations [22] by having a positive effect on the maintenance of Nanog levels. When the level of LIF decreases, the cells will differentiate (figure 3). Similarly, i2/i3 which are represented by *I*_3_ in our propensity functions, are two different sets of small molecule inhibitors found to be capable of maintaining pluripotency *in vivo* [23]. In our model, increase of I3 will lead to the suppression of Fgf4, which in turn reduces the effect of suppression on Nanog and therefore sustains the pluripotent state. The model includes the possibility of reprogramming by introducing signal *α* in the second propensity function. It can be interpreted as the reprogramming rate of the backward transition from the differentiated state back into the pluripotent state: when *α* reaches a critical value, Oct4-Sox2 will induce Nanog and keep raising it towards sufficiently high value and in consequence it will reduce Gata6 level by antagonism [20]. For simplicity, here we only consider the effects of LIF; I3 and *α* are assumed to be zero throughout this analysis. The level of LIF was chosen as fifty for both reference data and simulations in ABC–SMC.

#### 2.3.1. Simulation procedure

Equation (2.4) can be expressed in the form of a chemical reaction system [24]:

2.6 $$\frac{dx(t)}{dt}=S\nu (x(t)),$$

where **S** is the stoichiometric matrix and *ν*(**x**(*t*)) is the reaction rate vector.

However, ODEs in this form neglect the stochastic nature of the cell differentiation systems. To incorporate the stochastic nature, we could consider the corresponding SDE (equation (2.2)). Here, instead we include the stochastic dynamics by considering the chemical master equation description [25]:

2.7 $$\frac{dP(x,t|x_{0},t_{0})}{dt}=\sum_{i=1}^{M}[a_{i}(x)P(x-\nu_{i},t|x_{0},t_{0})-a_{i}(x)P(x,t|x_{0},t_{0})],$$

where *M* is the total number of reactions and *a*_*i*_(**x**) = *a*_*i*_(**x**(*t*)) is the propensity function for the *i*th reaction. This chemical master equation can be thought of as allocating probability distribution to the trajectory of ODE in equation (2.4). We obtain the simulated results by solving the chemical master equation through the Gillespie algorithm [26].

We note that when assuming *a*_*i*_(**x**) remains constant on time interval [*t*, *t* + *τ*] for some time step *τ* > 0, the solution to equation (2.7) is equivalent to solving the following SDE [25] with the propensity function as strength of multiplicative noise:

2.8 $$dx(t)=\sum_{i=1}^{M}\nu_{i}a_{i}(x)\;dt+\sum_{i=1}^{M}\nu_{i}\sqrt{a_{i}(x)}\;dW_{i}(t).$$

In practice, it may be impossible to obtain suitable time course experimental observations. Instead, experimental observations are given as snapshots covering cells at different stages of development or differentiation. To generate our reference data, we sample cells at 10 equally spaced time points. This leads to a dataset that mimics aspects of real experiments [27,28].

Our ABC–SMC procedure is adapted to this sampling scheme. Specifically, we generated our reference data with sample size 300 by the true parameters in equation (2.5). Each sample has a time-dependent trajectory with four different transcription factors as in equation (2.4). We then chose 10 evenly distributed time points for each trajectory and this would lead to a 4 × 10 × 300 matrix as reference data (4 factors, 10 time points and 300 trajectories). Under the ABC–SMC scheme, test data are simulated in the same way where the parameters of the model are now based on the current candidate parameters. The distance is obtained by summing over all 10 time points (i.e. $d=\sum_{i=1}^{10}\;d(D_{i}^{*},D_{i})$, where *D*_*i*_ and $D_{i}^{*}$ are 4 × 300 matrices).

For the stochastic simulations (using the Gillespie algorithm [26]), we use the Julia packages *Catalyst.jl* and *DifferentialEquations.jl*. For the ABC–SMC algorithm, we use *ApproxBayes.jl*.

We choose the sequence of tolerance threshold $\epsilon_{t}$ based on the 30% quantiles of the population of particles in the last iteration until it reaches the target tolerance threshold $\epsilon_{T}$, or until it exceeds the set maximum number of simulations (10^7^). That is, $\epsilon_{t}=q_{\alpha =0.3}(\theta_{t-1}),t\in [1,T]$. For kernel density estimation (KDE) with multivariate normal distributions, we use *KDEstimation.jl* and we set the bandwidth according to Silverman’s rule. The computation of optimal transport (OT) with Sinkhorn divergence algorithm [29] is performed in *OptimalTransport.jl*.

**Figure 4.**
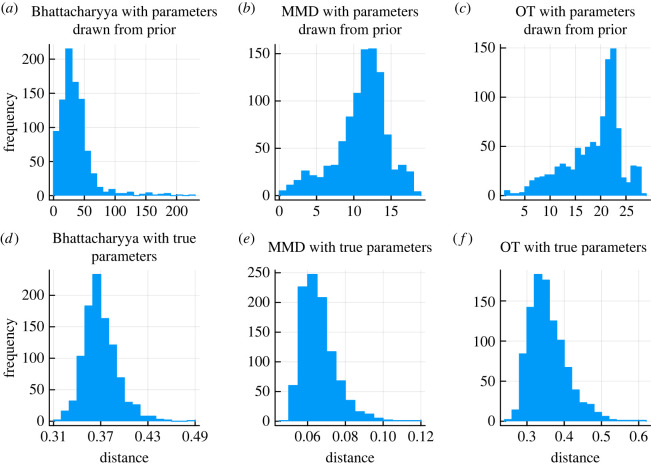
The distribution of distances of 1000 comparisons for different distance metrics. (*a*) Bhattacharyya distances with parameters drawn from the priors. (*b*) MMD with parameters drawn from the priors. (*c*) Optimal transport by Sinkhorn divergence algorithm with parameters drawn from the priors. (*d*) Bhattacharyya distances with identical parameters. (*e*) MMD with identical parameters. (*f*) Optimal transport by Sinkhorn divergence algorithm with identical parameters.

For all parameters, uniform prior distributions, with lower bound given by 1/10 of the true parameters in equation (2.5) and upper bound given by 10 times the true parameters, are used. Since all the data are acquired by simulations rather than from real experimental results, we can choose the final tolerance threshold $\epsilon_{T}$ by simulating data with exactly the same parameters and comparing the distance between them. To ensure our distance metric can distinguish the true parameters from the simulated data, we ran 1000 simulations for the true parameters and 1000 with parameters drawn from the priors for each of the distance metrics. All the comparisons are summarized in the density plot, in figure 4. We can see that for each metric, the distributions barely overlap (note the significant difference in scale for each metric). Therefore, few incorrect particles will be chosen and few correct particles will be rejected when the threshold $\epsilon_{T}$ is sufficiently small around the neighbourhood of the distance distributions for the true parameters. We therefore chose specific $\epsilon_{T}$ values for each distance metric based on these results to correspond to the central of the density obtained for the true parameters. In practice, choice of the $\epsilon_{T}$ schedule is difficult and can affect convergence and speed of convergence considerably [30]; it also depends crucially on the choice of distance metric, and below we discuss a range of suitable distance metrics for single-cell data and their relevance for inferring the parameters of models of cell differentiation.

## 3. Results

### 3.1. Distances for single-cell data

In equation (2.3), we determine the quasi-potential $\tilde{U}(X)$ from the probability distribution over cell states, *X*. For our ABC–SMC procedure, we need suitable distance measures between probability distributions. In this section, we examine and compare some measures that quantify the discrepancies between probability distributions, as well as their underlying geometric properties before adopting them in our ABC–SMC inference scheme.

#### 3.1.1. KL-divergence

We start with the KL-divergence, which is one of the simplest and most commonly used measures in comparing probability distributions. For data *D* ∼ *Q* and model distribution $P_{\theta }$, the KL-divergence is defined as

3.1 $$d_{KL}(P_{\theta },Q)=\int \;q(x)\log\left(\frac{q(x)}{p_{\theta }(x)}\right)\mu (dx).$$

Here, *q* and $p_{\theta }$ are the corresponding probability density functions with respect to the same probability measure *μ*. The KL-divergence is closely related to maximum-likelihood theory [31]:

3.2 $$\theta_{\min\;KL}=\underset{\theta }{\mathrm{argmax}}\frac{1}{n}\sum_{i=1}^{n}\log P_{\theta }(x_{i})=\underset{\theta }{\mathrm{argmax}}\log p_{\theta }(x)=\underset{\theta }{\mathrm{argmax}}p_{\theta }(x)=\theta_{\mathrm{MLE}}.$$

However, if there exist *x*_*i*_ such that *q*(*x*_*i*_) = 0, the KL-divergence diverges to infinity, which can become problematic in numerical computations. Because of the numerical issues, we prefer other measures of the distances between probability distributions.

#### 3.1.2. Bhattacharya distance

The Bhattacharyya distance is used in signal processing and pattern recognition [32] and defined by

3.3 $$B(P,Q)=-\log\left(\int \sqrt{q(x)p(x)}\;dx\right)=-\log(\rho ),$$

for distributions *P*, *Q* with probability density functions *p*(*x*) and *q*(*x*); $\rho (P,Q)=\int \sqrt{\;p(x)q(x)}\;dx$ is also known as the Bhattacharyya coefficient. Bhattacharyya is unbounded (0 ≤ *B* ≤ ∞) and unlike the KL-divergence, it is a symmetric metric.

An advantage of the Bhattacharya distance is that it has been proved that for any two sets of parameters *θ*_1_, *θ*_2_, if we have *B*(*θ*_1_) > *B*(*θ*_2_) then there exist *π* = (*π*_1_, *π*_2_) prior distributions that satisfy *P*_*e*_(*θ*_1_, *π*) < *P*_*e*_(*θ*_2_, *π*), where *P*_*e*_(*θ*, *π*) is the error probability with parameter set *θ* and prior probability *π* [33]. This property makes it particularly meaningful for parameter estimation in an ABC framework by considering the optimal Bayes error probabilities. This metric gives good results for Gaussian noise problems, which may be useful for SDE problems that use standard Brownian noise [32].

There are two drawbacks shared by the KL-divergence and the Bhattacharyya distance:

(i) Our model is implicit (i.e. the data points are generated by a set of equations describing the biophysical processes), but the corresponding distributions cannot be represented by a density function without using KDE or a different density estimation methods in high-dimensional space. However, due to the curse of dimensionality, the number of data points required to have accurate estimates increases dramatically with increasing dimensionality [34]; distance metrics which require KDE would be problematic not only in terms of accuracy but also efficiency.
(ii) This metric only measures the difference of probability density in a point-wise way which ignores the underlying geometry of the probability space [35].

Therefore, we next introduce distance metrics that do not require KDE and which take the geometry of feature space into account.

#### 3.1.3. Optimal transport

Next, we consider an OT distance metric that is free of density estimation by comparing samples directly and maintaining the underlying geometry in feature space. This metric has been widely used in the field of machine learning and image recognition for its efficiency [36]. Intuitively, the key to OT is to figure out the minimal cost required to transform one image into another, or in our case, one probability distribution into another.

Suppose we have two probability distributions, *P*, *Q*, on the probability space, χ, *Γ*, with probability measures, *μ*, *ν*, respectively. Let $T_{\#}\mu$ denote the forward of *μ* by *T*:

$$T_{\#}\mu (B)=\mu (x\;:\;T(x)\in B)=\mu (T^{-1}(B)),\;B\in \sigma (\Gamma ).$$

The original version of OT is defined in [37]:

3.4 $$\underset{T_{\#}\mu =\nu }{\inf}\int c(x,T(x))\mu (dx),$$

where c : χ×Γ↦[0,∞) is a measurable function on χ×Γ known as the cost function.

However, this technique is clearly not applicable when there does not exist any forward equation *T* such that $T_{\#}\mu =\nu$. To overcome this, we adopt Kantorovich’s version of OT or the *p*-Wasserstein distance *W*_*p*_(*P*, *Q*) with Euclidean distance as cost function, defined as [37]

3.5 $$W_{p}(P,Q)={\left(\underset{\pi \in \Pi (P,Q)}{\inf}\int \|x-y{\|}^{p}\;d\pi (x,y)\right)}^{1/p},\;p\geq 1,$$

where Π(*P*, *Q*) represents all the joint probability measures on χ×Γ with marginal measures, *μ* and *ν*, respectively. This version is known to be well defined in general scenarios [37].

If in addition we have $f_{P}\;:\;\chi \mapsto R$ and $f_{Q}\;:\;\Gamma \mapsto R$ bounded and measurable functions with

3.6 $$f_{P}(x)-f_{Q}(y)\leq c(x,y),$$

it is easy to formulate the duality form of OT [38]:

3.7 $$W_{p}{\left(P,Q\right)}^{p}=\underset{\;f_{P},f_{Q}}{\sup}\left(\int f_{P}(x)\mu (dx)-\int f_{Q}(x)\nu (dy)\right).$$

Since we are working on the same probability space for both *P* and *Q*, say χ=Γ, we can have the generalized 1-Wasserstein under 1-Lipschitz (Lip1) continuous functions on χ [39]:

3.8 $$W_{1}(P,Q)=\underset{\;f\in Lip1}{\sup}\left(\int f\;(x)P(dx)-\int f\;(x)Q(dx)\right).$$

If we have two different probability distributions, *P*, $Q\in P_{\chi }$, then the mixture distribution

3.9 $$P_{t}=(1-t)P+tQ,\;\forall \;t\in [0,1],$$

can be considered as a curve connecting *P*, *Q* under the whole space of probability distributions, $P_{\chi }$. It can be shown that for *p* = 2, the Wasserstein distance will give a minimal geodesic under such a probabilistic space of distributions [39]. In other words, whenever OT proposes a probability distribution that transports from *P* to *Q* under χ, we will have the shortest path or distributions between *P* and *Q*. This result gives a meaningful insight in our ABC–SMC framework and allows us to compare samples and simulations geometrically. It is worth noting that there may exist many minimal geodesics given by the OT which satisfy equation (3.9).

#### 3.1.4. Maximum mean discrepancy

The maximum mean discrepancy (MMD) measure has been used for of goodness of fit tests [40], but also in an ABC framework [41]. The principle here is to find a kernel function that will give different expected values from two samples when they are not drawn from the same distribution. The underlying rationale is that the evaluations of such function at sample points, which are drawn from different probability distributions, can provide enough information about their difference [42].

The initial definition of MMD is as follows. Let F be a class of functions f : χ↦R and *P*, *Q* be distributions of samples from *X*, *Y* which are independent and identically distributed with respect to *P* and *Q*; then MMD can be defined as

3.10 $$\text{MMD}(F,P,Q)=\underset{\;f\in F}{\sup}(E_{P}[f\;(x)]-E_{Q}[f\;(y)]).$$

It is clear that if and only if *X*, *Y* are drawn from the same distribution (i.e. *P* = *Q*) then the MMD is zero for any functions f∈F; but if the class of functions F is too ‘large’, the statistic would be much greater than 0 for most of the finite samples *X*, *Y*, which reinforces the differences in distribution.

In order to avoid this scenario while simultaneously allowing reasonable discrepancies between P and Q to be detected, there must be a suitable restriction on the class of function F. The trade-off is achieved by restricting F to be the unit ball in reproducing kernel Hilbert space (RKHS) [42]. The MMD can be calculated from this space by calculating the expected values of this kernel function under distributions *P*, *Q*. Let H be a RKHS on feature space χ induced by a feature map ϕ : χ↦R. The kernel is given by the inner product between the feature maps under RKHS:

3.11 $$k(x,y)\;:={\left\langle \phi (x)\;\mathrm{and}\;\phi (y)\right\rangle }_{H}.$$

It is guaranteed that MMD can detect differences between distributions *P*, *Q* of samples from *X*, *Y*. When F is restricted to the unit ball in the RKHS (i.e. $F\;:=\;f\;:\;\chi \mapsto R|\|f{\|}_{H}\leq 1$), then MMD(F,P,Q)=0 if and only if *P* = *Q* [42]. With characteristic kernels (i.e. kernel mapping is injective), MMD is proven to be a probabilistic metric which takes values in distribution functions [43]. A standard choice of this is the Gaussian kernel:

3.12 $$k(x,y)=e^{{\left(x-y\right)}^{2}/2\sigma^{2}}.$$

We will use the median heuristic to set the bandwidth of Gaussian kernels with *σ* = median(*X*, *Y*)/2 [44].

In practice, given samples from *X* with sample size *m*, and from *Y* with sample size *n*, it is straightforward to determine the corresponding MMD:

3.13 $$\begin{array}{rl} {\mathrm{MMD}}^{2}(F,X,Y) & =\frac{1}{m(m-1)}\sum_{i\neq j}^{m}k(x_{i},x_{j})\\ & \;+\frac{1}{n(n-1)}\sum_{i\neq j}^{n}k(y_{i},y_{j})-\frac{2}{mn}\sum_{i=1}^{n}\sum_{\;j=1}^{m}k(x_{i},y_{j}). \end{array}$$

Since MMD can be computed from empirical observations, it can be adapted to ABC–SMC simulators.

MMD is defined similarly to OT (3.8) under different sets of functions F. Such metric problems are known as integral probability metrics (IPMs) [45]. It has been shown that all such IPMs preserve the minimal geodesic property and it has further been proved that there exists a unique minimal geodesic under MMD in comparison to OT (for details, see theorem 5.3 in [39]).

### 3.2. Approximate Bayesian computation inference depends on distances

The obtained approximate posterior distribution of all the parameters for each distance metric are summarized in figures 8–10. Our results of posterior distributions suggest a good agreement in estimating parameters using ABC–SMC with different metrics. With regard to the identifiability of parameters, we observe well-structured shape of the posterior probability distributions for most of the parameters (*c*_1_, *c*_2_, *c*_3_, *e*_1_, *b*_1_, *b*_2_, *b*_3_); other parameters are more likely to have ‘flat’ distributions and are therefore not well inferable.

**Figure 5.**
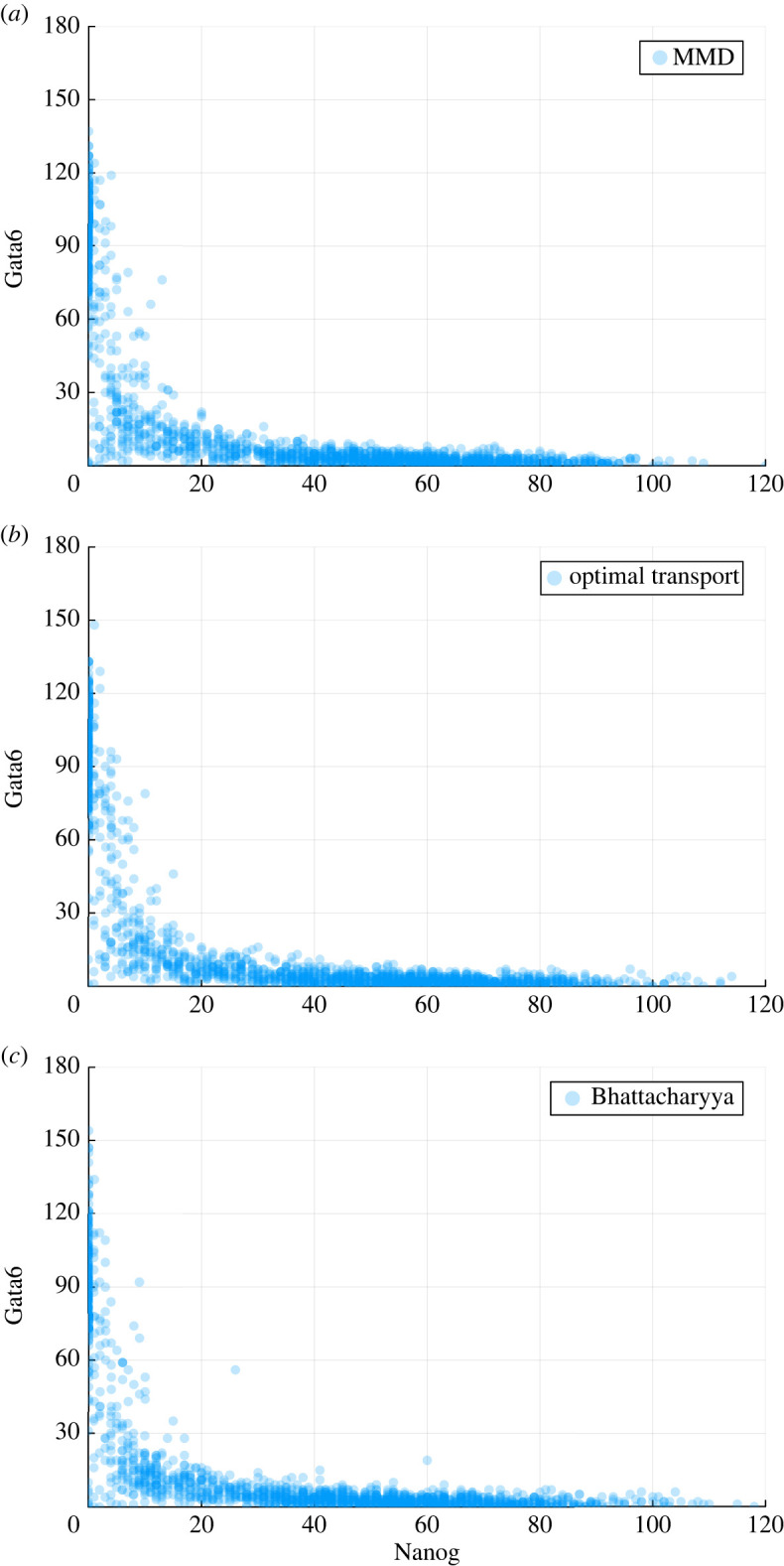
The plot of Nanog versus Gata6 at final time point using sampled particles from the last population. (*a*) Simulated population using sampled parameters from MMD, (*b*) simulated population using sampled parameters from optimal transport by Sinkhorn divergence, (*c*) simulated population using sampled parameters from Bhattacharyya distance.

Here, we test the plausibility of the estimated posterior distributions by drawing particles from such posterior distributions and see if we can reproduce the dynamic trajectories of our system. The simulation results using ABC–SMC posterior samples with different distance metrics are given in figure 5; and they are similar is to the dynamics as in reference data (figure 3*b*). Our result suggests both the applicability of ABC–SMC in parameter estimations from single-cell data and the importance of choosing an adequate distance measure.

We choose the sequence of tolerance thresholds for MMD as the 30% percentile, which gives rise to a schedule of

3.14 ϵ∈{0.34,0.78,0.28,0.15,0.10,0.07,0.06}.

See figure 4. For comparison, the sequence of tolerance thresholds for OT is

3.15 ϵ∈{4.61,2.72,1.15,0.75,0.59,0.5,0.46},

which decreases constantly even after reaching the boundary support of density for true parameters in figure 4. Our result indicates that in an ABC–SMC context OT may lead to more efficient parameter inference than MMD.

The ABC–SMC posterior distribution allows us to analyse relationships among the parameters in our system. We can determine underlying correlations between pairs of parameters in the pair-wise plots (figure 8). We find that *c*_1_ and *c*_3_ are strongly correlated with a clear linear trend and some slight linear relationships are also shown between *e*_1_ and *e*_2_ and *b*_2_ and *b*_3_. With regards to the identifiability of relationships between *b*_2_ and *b*_3_, we find that the Bhattacharya distance lags behind those of other distances (figure 10).

Many dynamical systems, including biological ones, are known to exhibit ‘sloppy’ behaviour: the dynamical behaviours of such systems are usually controlled by a small number of parameters [46,47]. In our results, see figures 8–10, it would, for example, be easy to determine the value of *c*_3_ or *c*_1_ if the other one is known while reproducing the same trajectories of transcription factors due to the correlation between them. We will show that our approach can help resolve this problem by identifying such sets of correlated parameters from joint distribution and the detail of this ‘sloppy’ analysis will be discussed in the following section.

With regard to computational cost, we found that MMD is the fastest one and OT follows as explained by the theoretical time complexity [48]. Inference with the Bhattacharya distance takes more than three times longer compared to MMD, due in no small measure to the time taken up by KDE density estimation. This reinforces the need for methods that do not require density estimation in our ABC–SMC framework for single-cell analysis.

### 3.3. Parameter sensitivity

Visualizing and analysing the sensitivity of parameters is challenging even from our joint (approximate) posterior distributions. The simplest and most convenient way is to directly focus on the variance of the posterior distributions. We therefore adopt principal component analysis (PCA) to measure the variability of parameters [49,50].

Starting with the posterior distributions of our parameters obtained from ABC–SMC, we can use the traditional method of moments to estimate the sample covariance matrix Σ:

3.16 $$\Sigma =\frac{1}{n}\sum_{i=1}^{n}(X_{i}-\bar{X}){\left(X_{i}-\bar{X}\right)}^{T}.$$

We estimate the variance of each parameter after applying the spectral decomposition to Σ:

3.17 $$Q^{T}\Sigma Q=\text{diag}(\lambda_{1},\lambda_{2},\ldots ,\lambda_{p}),$$

where *Q* = (*q*_1_, …, *q*_*p*_) is the eigenparameter (vector). The eigenvectors form an orthogonal matrix and *λ*_1_, *λ*_2_, …, *λ*_*p*_ are the corresponding variances of each parameter which are known as the principal components of Σ. Therefore, we can measure the variability of data explained by each parameter by diagonalizing the covariance matrix and performing the sensitivity analysis.

If *q*_*j*,*k*_ is the direction associated with the *k*th parameters then we will have the projection of *θ*_*k*_ through the inner product,

3.18 $$c_{\;j,k}=q_{\;j,k}\cdot \theta_{k},$$

where *c* = (*c*_1_, …, *c*_*p*_) are such principal components:

3.19 $$\begin{array}{rl} \text{cov}(\theta_{k},c_{j}) & =\text{cov}(q_{\;j,k}\cdot \theta_{k},c_{j})\\ & =q_{\;j,k}\lambda_{k}, \end{array}$$

which gives the correlations between principal components *c*_*j*_ and eigenparameters *θ*_*k*_. Thus, we can use the standardized square correlations,

3.20 $$\rho_{\theta_{k},c_{j}}^{2}=\frac{q_{\;j,k}^{2}\lambda_{k}}{\sum_{i=1}^{p}q_{i,k}^{2}\lambda_{i}},$$

to interpret the proportion of variance eigenparameter *θ*_*k*_ explained by principal component *c*_*j*_. From this, we can use the total proportion of variability explained by parameter *θ*_*k*_ to quantify the sensitivity of parameter *θ*_*k*_ by

3.21 $$s_{k}=\sum_{i=1}^{p}\rho_{\theta_{k},c_{i}}^{2}.$$

We note that using PCA to analyse sensitivity is the same as eigenvalue analysis of the Hessian matrix around the MLE or the equation of least-square errors [50].

We can define the log posterior density by

3.22 h(θ)=log⁡(P(θ|D))=log⁡(P(D|θ))+log⁡(π(θ)).

And using Taylor expansion of logarithm along with the log posterior distributions, we obtain [50]

3.23 $$h(\theta )=h(\hat{\theta }){\left(\theta +\hat{\theta }\right)}^{T}h^{\prime \prime }(\hat{\theta })(\theta -\hat{\theta })+O(\theta^{3}),$$

where $h^{\prime \prime }(\hat{\theta })$ is the Hessian matrix of log posterior distribution.

When assuming the parameters are drawn from the multivariate normal distribution, we can deduce the asymptotic covariance matrix of *θ* by the Fisher information matrix:

3.24 $$\text{Var}[\theta ]={\left[-h^{\prime \prime }(\hat{\theta })\right]}^{-1}.$$

From this, we can conclude that the eigenvalues of the variance-covariance matrix *λ*_*k*_ in equation (3.17) and the eigenvalues of the corresponding Hessian matrix *v*_*k*_, which results in asymptotic variance by assuming multivariate normality, are inversely related [51]:

3.25 $$\lambda_{k}=\frac{1}{v_{k}},$$

which legitimates the statistical property of our PCA result.

**Figure 6.**
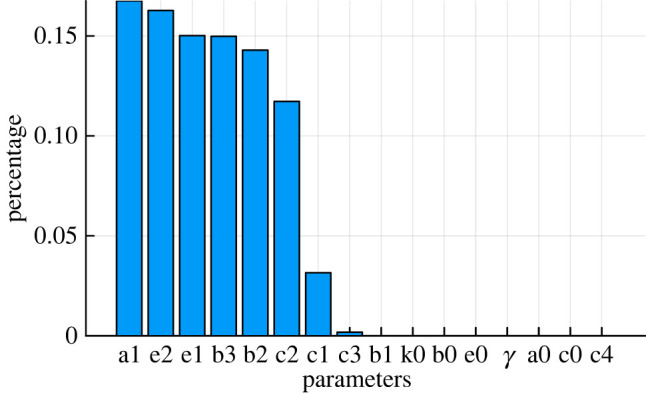
Parameter sensitivity for the stem cell model. Where the percentage of sensitivity for each parameter stands for the total proportion of model variability explained by parameter *θ*_*k*_ which is quantified by the sum of covariance of parameter *θ*_*k*_ between all the principal components obtained from PCA on parameter space (see equations (3.20) and (3.21)).

We summarize the result of variability/sensitivity of each parameter in figure 6. It is known that change of value in sloppy parameters will alter the system behaviours only slightly, while similar change of value in stiff parameters will lead to pronounced changes in dynamics. In terms of parameter variance, if we characterize parameters that explain more than 1% of the variance for the posteriors as sloppy and otherwise as stiff we can conclude that parameters *a*_1_, *b*_2_, *b*_3_, *c*_1_, *c*_2_, *e*_1_, *e*_2_ are sloppy parameters and inferred with higher uncertainty.

**Figure 7.**
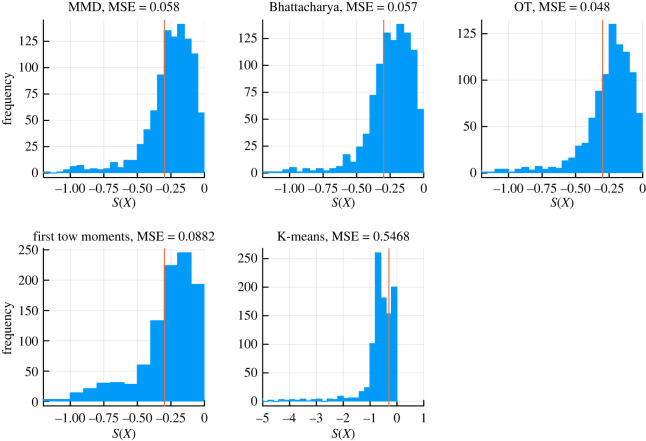
Realized versus posterior predictive distributions for ABC–SMC results obtained with the use of different distance metrics: S(X)=log⁡( p/(1−p)) (where *p* is the probability a stem cell ends in pluripotent state), compared to simulations from the posterior predictive distribution for each distance metric. The vertical line presents the test statistics from simulation of true parameters and the corresponding mean squared errors (MSEs) for different metrics are presented.

Our result suggests that all the regeneration rates (*c*_0_, *e*_0_, *a*_0_, *b*_0_), as well as the constant degradation rate *γ*, are stiff. This is not surprising since these parameters determine the extent of change in each factor over the whole time course. For the transcription factors Oct4-Sox2 and Fgf4, all other parameters are sloppy, except for their respective regeneration rates and the degradation rate *γ*, which explains the dynamics in agreement with previous analyses [16]. In view of the scaling parameters *k*_0_, *c*_4_ for Oct4-Sox2 and Gata6 as inputs into Nanog, we find that the effects of Oct4-Sox2 and Gata6 on Nanog, and hence on the fate decision making in our system are very distinct. By contrast, the effect of Nanog on Gata6 is less constrained, based on the sloppy scaling parameter *b*_2_. The self-regeneration or degradation rate *b*_1_ is much more pronounced for Gata6.

## 4. Discussion

The inference of parameters from experimental single-cell data for complex systems is of great practical interest. A range of inference frameworks have been proposed [51–55], and in ABC settings summary statistics coupled with simple distance metrics, like the Euclidean distance or *L*^*p*^ norm [56].

Recent studies, inspired by Waddington’s landscape, have shown that the quasi-potential can be used quantitatively [16–18,21]. But to put this into an inferential framework requires some care, including identification of suitable distance metrics over the quantitative gene expression measurements.

In this study, we investigate parameter inference through the ABC framework using different distance metrics. We explore the effectiveness of three metrics: MMD, Bhattacharya distance, and OT by Sinkhorn divergence algorithm. Our results show that the inference of parameters based on these metrics can successfully capture the dynamics of a stem cell differentiation model.

To highlight the usefulness of the quasi-potential landscape in determining model parameters within the ABC framework, we conducted a comparison by employing our ABC–SMC algorithm with the simple Euclidean distance metric. Specifically, we considered two types of summary statistics: the first and second moments (*E*(*X*), *E*(*X*^2^)) and the logarithm-transformed probability of cells in pluripotent states (log⁡( p/(1−p))), where *p* was determined using K-means with two classes.

**Figure 8.**
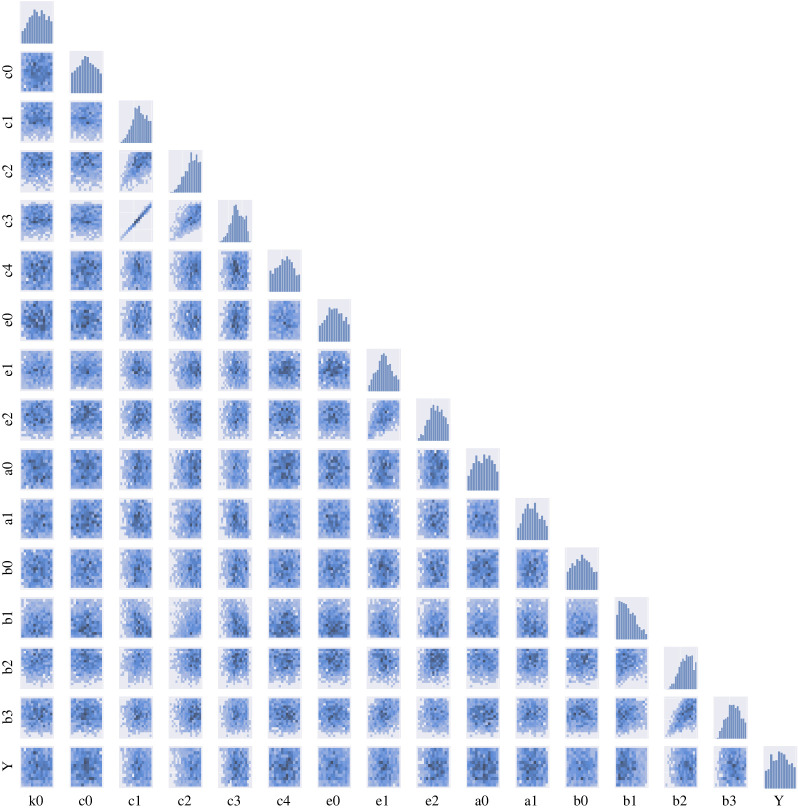
Results of ABC–SMC in parameter estimations for the stem cell model using optimal transport by Sinkhorn divergence. Diagonal: histogram of each parameter. Lower triangle: two-dimensional pairwise scatter plot of the joint posterior distributions between two parameters.

**Figure 9.**
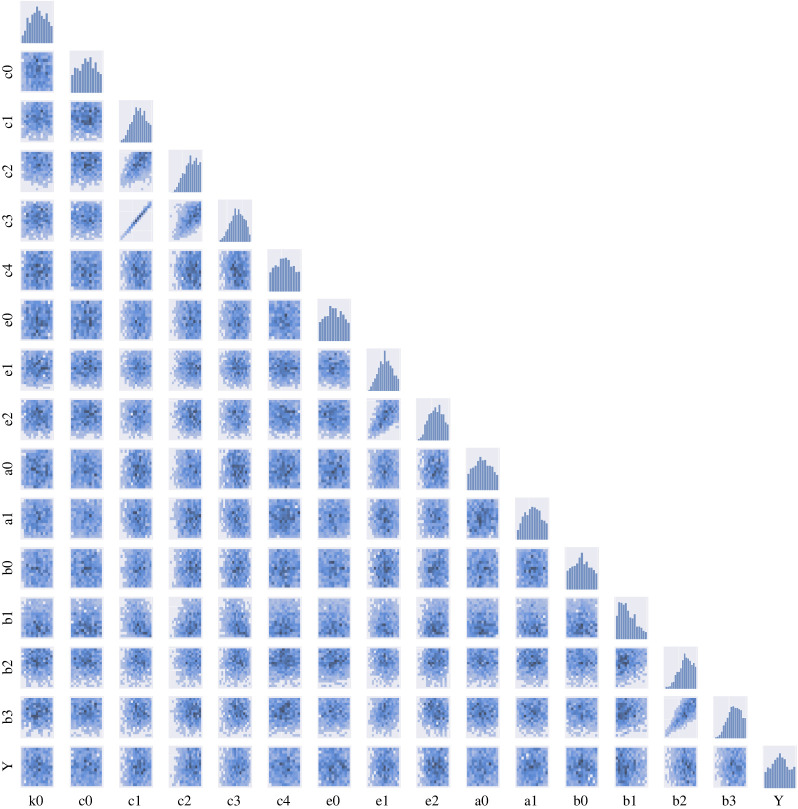
Results of ABC–SMC in parameter estimations for the stem cell model using MMD, similar to figure 8.

**Figure 10.**
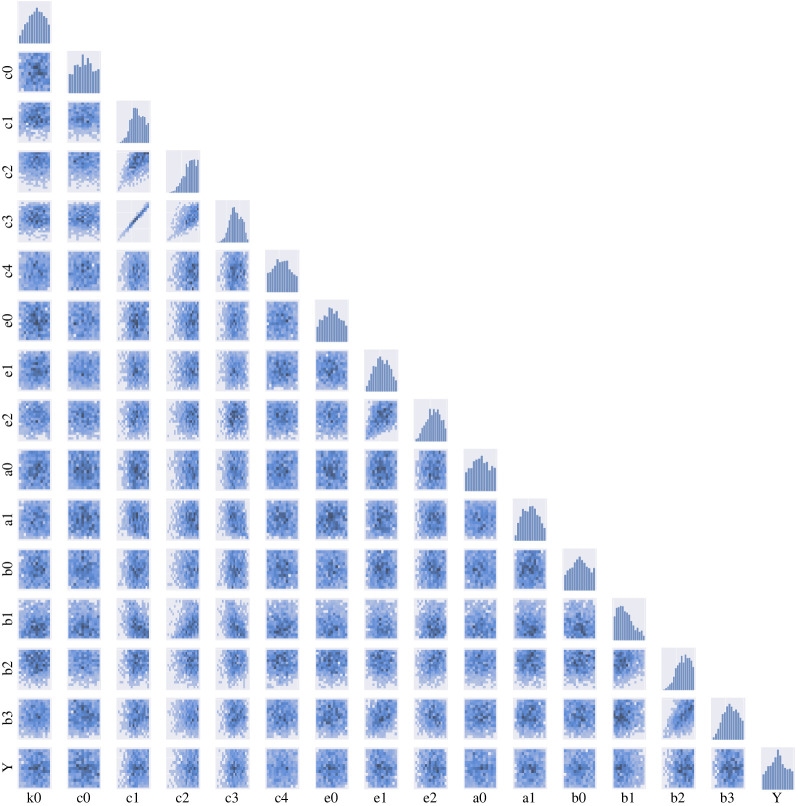
Results of ABC–SMC in parameter estimations for the stem cell model using Bhattacharya distance, similar to figure 8.

We summarized the results of the posterior predictive distribution check using S(X)=log⁡( p/(1−p)) as the test statistic (figure 7). We outlined the extreme quantity *p* (*Pr*(*S*(*X*) > *S*(*D*))) and mean squared value (MSE) and observed that the posterior distributions obtained using distance metrics that compare probability distributions were more consistent with the simulated experimental data, outperforming those obtained with the Euclidean distance. Therefore, we concluded that accessing information from the landscape is crucial for determining the parameters of dynamical systems. To achieve this, it is necessary to adopt distance metrics capable of quantifying the differences between probability distributions.

Our findings suggest that among the evaluated metrics for comparing distributions, the Sinkhorn divergence yields the best performace in ABC inference. It is known that Sinkhorn divergence and MMD belong to the same category of metrics known as IPMs [45]. When they both use the same kernel functions, the Sinkhorn divergence can be considered as an interpolation of MMD [48]. We suggest that Sinkhorn divergence would perform better in determining the target estimate, especially when employing gradient flows such as in the gradient descent approach [35]. Our study further suggests that Sinkhorn divergence also has advantages for applications in non-gradient approaches, like in the ABC scheme.

With regard to computational cost, both MMD and Sinkhorn divergence metrics are similar ($O(N^{2}d)$ for MMD [42] and $O(CN^{2}\log N)$ for Sinkhorn divergence [57], where *N* is the sample size, *d* is the dimensionality and *C* is a constant depending on the input matrices). They both outperform the Bhattacharya distance. The key reason for the computational difference with the Bhattacharya distance lies in the estimation of the KDE density ($O(Nm^{d})$ where *m* is number of resampling points on each dimension [58]). This drawback will become more limiting as the dimensionality increases.

Considering the computational differences, we recommend using MMD as the distance metric where samples are directly compared without density estimation and implicitly quantifying the differences between distributions. This property makes MMD computationally efficient and well-suited for dealing with high-dimensional data and it offers a more practical and efficient option for parameter inference with ABC algorithm in dynamical systems.

Although the potential landscape offers valuable insights into the dynamics of biological systems, it is not the sole factor governing these processes. Conventionally, the quasi-potential is defined by taking the negative logarithm of the probability distributions, assuming a static and time-homogeneous landscape [59]. However, recent research has delved deeper into this field, revealing that cell systems are time-inhomogeneous, and highlighting the existence of transient landscapes [16,21,60,61].

To capture the time evolution of stem cells accurately, it is essential to consider the curl of probability flux, which interacts with the potential landscape to govern the ongoing trajectories of cell fates [62]. In our approach, although we attempted to quantify the variational structure of the landscape by collecting data points at different times, we still lost the information related to the probability flux.

Since we have demonstrated the significance of landscape information in inferring parameters from single-cell data, we believe that our framework can be further enhanced. Incorporating and measuring the probability flux under appropriate distance metrics is one such potential improvement. This extension would allow us to gain a more comprehensive understanding of the underlying dynamics and improve the accuracy of parameter inference in complex systems.

## Data Availability

All data and code used in the study are open source and freely available via https://github.com/YUJINGl3/Approximate-Bayesian-Computation-for-Inferring-Waddington-Landscapes-from-Single-Cell-Data.git and have been archived within the Zenodo repository: https://doi.org/10.5281/zenodo.11100350 [63].
